# Towards Personalized Medicine: An Improved *De Novo* Assembly Procedure for Early Detection of Drug Resistant HIV Minor Quasispecies in Patient Samples

**DOI:** 10.6026/97320630014449

**Published:** 2018-09-18

**Authors:** Cindy Huang, Vichetra Sam, Sophie Du, Tuan Le, Anthony Fletcher, William Lau, Kathleen Meyer, Esther Asaki, Da Wei Huang, Calvin Johnson

**Affiliations:** 1 Center for Information Technology, National Institutes of Health, Bethesda, Maryland 10891; 2 Thomas Wootton High School, Rockville, Maryland 20850; 3 CSRA, Falls Church, VA 22042

**Keywords:** *De Novo* Assembly, HIV, PacBio, quasispecies

## Abstract

The third-generation sequencing technology, PacBio, has shown an ability to sequence the HIV virus amplicons in their full length. The long read of PaBio offers a distinct advantage to comprehensively understand the virus evolution complexity at quasispecies level (i.e. maintaining linkage information of variants) comparing to the short reads from Illumina shotgun sequencing. However, due to the highnoise nature of the PacBio reads, it is still a challenge to build accurate contigs at high sensitivity. Most of previously developed NGS assembly tools work with the assumption that the input reads are fairly accurate, which is largely true for the data derived from Sanger or Illumina technologies. When applying these tools on PacBio high-noise reads, they are largely driven by noise rather than true signal eventually leading to poor results in most cases. In this study, we propose the de novo assembly procedure, which comprises a positivefocused strategy, and linkage-frequency noise reduction so that it is more suitable for PacBio high-noise reads. We further tested the unique de novo assembly procedure on HIV PacBio benchmark data and clinical samples, which accurately assembled dominant and minor populations of HIV quasispecies as expected. The improved de novo assembly procedure shows potential ability to promote PacBio technology in the field of HIV drug-resistance clinical detection, as well as in broad HIV phylogenetic studies.

## Background

HIV strains are frequently mutated from one HIV generation to the next, resulted in high genetic diversity of the HIV populations (named "quasispecies") in a given infected host over a time period [1, 2]. Particularly under certain selective pressure (e.g. antiretroviral treatment), certain HIV quasispecies with special characteristics (e.g. drug resistant, high transmission) could be propagated [1, 3]. Therefore, sequencing and analysis of the HIV quasispecies is important for improving personalized treatment plan, developing early prevention action, designing more effective vaccine for patients [1, 3, 4].

Even though the next generation sequencing (NGS) technology of Illumina shortgun sequencing offers improved variant detection ability (~1% detection limit) over the traditional Sanger-based sequencing method (~20% detection limit) [4, 5], both have the common weakness on sequencing HIV strains, that is, their detection of the mutations is at the individual mutation level and the important linkage relationship among mutations is lost during the procedure [6]. Furthermore, the recently emerging third generation sequencing, PacBio, can continuously sequence up to 10 kb for each read which in theory not only provides high detection sensitivity but also maintain the relationship of mutations [6, 7]. The PacBio technology shows the potential to change the HIV genetic study from individual mutation detection to explicit quasispecies detection [6, 7]. However, the high-noise nature of PacBio reads and lacks of effective data analysis tools still pose a barrier to fully utilize its power in the field of HIV genetics [6, 8]. Several works have addressed the challenges with different data analysis strategies, such as tag-focusing, error-correction, and clustering [4, 5]. Some shortages of these works include but are not limited to: 1. The error correction method heavily relies on certain mathematic assumptions concerning the errors in a certain statistical distribution, which may not be always held for all situations in reality due to complicated noise sources. 2. Most of the tools are reference-based approach, which could be a problem if sequenced sample is significantly different (i.e. large deletion; different HIV types) from wild type reference. 3. Most importantly, the error correction method might be over-trained on a simpler artificial training dataset without further cross-testing on real clinical patient samples, which latter could be much more complex and challenged than the artificial dataset. The unmet need in bioinformatics analysis requires further improvement of the algorithms.

In this report, we describe an improved de novo assembly procedure to accurately construct HIV quasispecies with high sensitivity. The PacBio read fasta file is the only required input without the need of reference sequence and other prior knowledge of the sequences. The procedure was successfully applied not only on HIV benchmark datasets, but also on real-life HIV relapse patient samples, leading to the early detection of the dynamic of HIV drug resistance strains.

## Methodology

### PacBio HIV benchmark data and clinical HIV patient samples

The HIV benchmark datasets and clinical patient data used in this report were adopted from a previously published paper [6]. In summary, two HIV strains (pLN4-3 and BN10) were admixed respectively with ratios of 90:10 and 99:1, and subjected to PacBio sequencing. In addition, two samples from the same HIV patient before and after treatment relapse were respectively sequenced using PacBio [6]. Approximately 15,000 to 20,000 CCS reads on the HIV POL region were obtained for each of the samples as described elsewhere [6]. Importantly, the prior known knowledge of actual mutation profiles of these samples can serve as references [6] to examine the results derived from the de novo assembly procedure.

### A unique de novo assembly procedure (Figure 1) to construct HIV quansipecies

#### PacBio CCS quality control

The quality of the CCS reads is typically associated with certain parameters of sequencing process [8]. We selected CCS reads with pass number >=3, average quality score >= 30 and minimum read length from 1000 bp to 1500 bp. In a typical situation, approximately 10% reads are filtered out by the above parameters. The post-QC CCR reads will be the only input of the de novo assembly analysis.

### Building consensus reference sequence(s) for the input data

To evaluate each CCR read, it is compared to an artificial consensus reference sequence(s) derived from the total input PacBio CCR reads. The consensus reference serves as real-time temporary standard for the given input dataset. The majority-rule based procedure [9] was used to build the consensus reference.

### Detecting all variant candidates

After building the consensus reference, each of the CCS reads was compared to it using the alignment program of BWA bwasw [10], which was design preferentially for long reads. After the alignment BAM file was generated, VarScan2 program [11] was used to select all variants with following criteria: substitution mutations; mutation read frequency >= 0.05%; not nearby a homopolymer region. As the CCR reads contain noise, the variants identified in this step are a mixture of true variants and noises.

### Selecting final variants (tags) with linkage-frequency base noise reduction algorithm

All possible paired combinations of the variant candidates from above step were formed. The actual co-occurrence frequencies of the pairs were surveyed by examining each of the CCR reads. The variant pairs with the actual co-occurrence frequency significantly beyond expected random co-occurrence frequency (Frequency of variant 1 x Frequency of variant 2) were determined by the G-test (see formula below), and selected in the final tag pool (Figure 2). In addition, single variant with high frequency (greater than 20%) was most likely true signal and selected to tag pool as well.

G = 2 ∑_i_ O_i_ · ln (O_i_ / E_i_)

where, Oi is the observed count and Ei is the expected count under the null hypothesis. In order assess the efficacy of the G test and determine the best cutoff value of the G statistic, performance data were assessed on the benchmark data set (Figure 3).

### Partitioning PacBio Reads based tag profiles into groups

Based on the final tag pool from above step, each CCR read have a mutation profile to represent its genetic character. The CCR reads were partitioned into groups based on the similarities of their mutation profiles. The number of groups is completely driven by the data itself ranging from 1 to n.

### Building final contigs for each PacBio read groups

Each of the PacBio read groups from above step represents a unique HIV quansispecies. The final contigs were built for each of the group using a previously reported clustering procedure [9].

### Automation of the de novo assembly procedure

**Figure 1:**
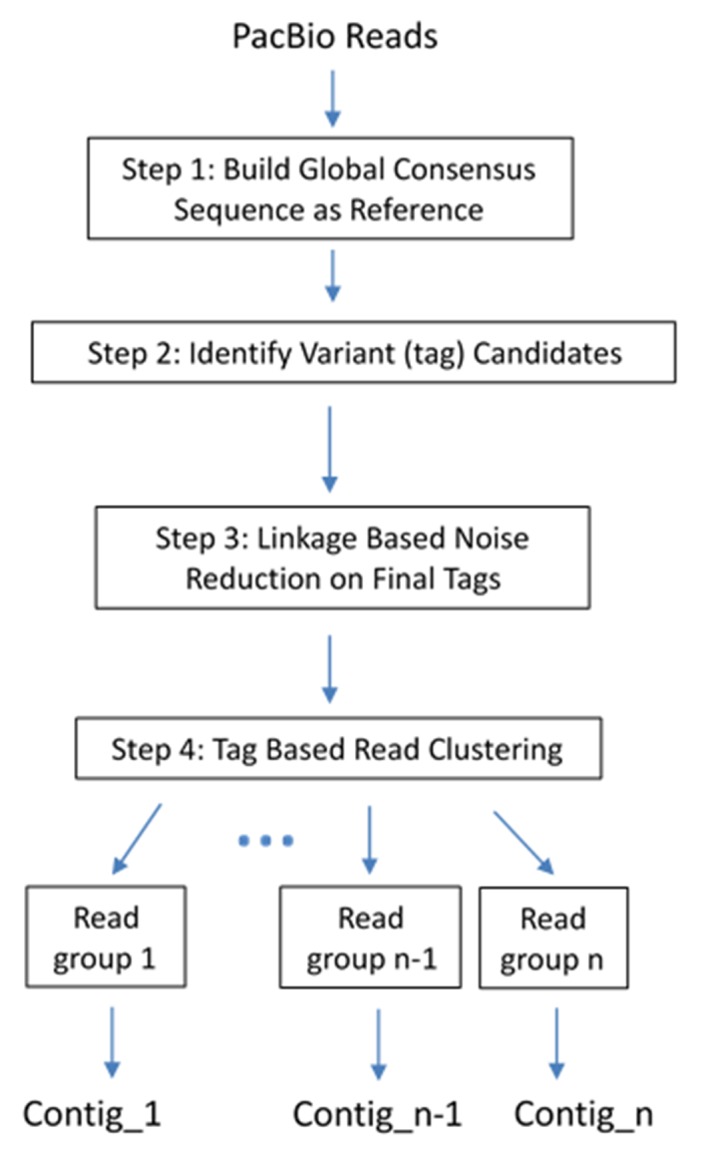
The sequential steps of the de novo assembly procedure. The procedure takes PacBio CCS reads as the only input data, and outputs final contig(s).

A Perl script was developed to automate above steps of the proposed de novo assembly (Figure 1). The only required input is a simple fasta file containing post-QC PacBio CCS reads. The script runs the input sequencing reads through each of the steps described in Section 2, and produces final contig sequences. It typically needs ~15-30 min to process a sample with ~20,000 CCR reads on a standard laptop computer or a Linux computer with 30 Gb memory and 1-4 CPUs. The automated script is available to the readers upon request.

## Results and Discussion

### The unique linkage-frequency based noise reduction plays a key role in the de novo assembly

**Figure 2:**
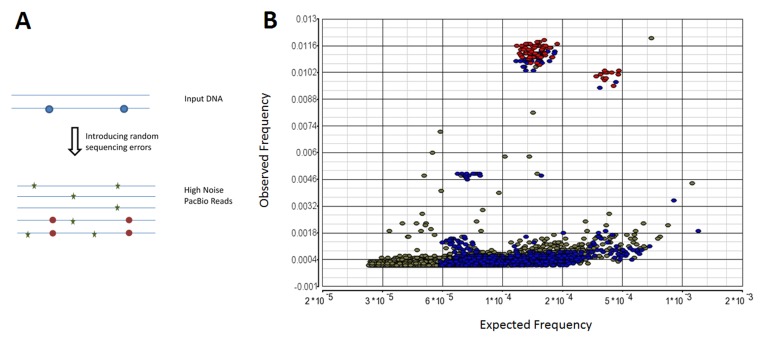
Linkage-frequency noise reduction. A. Freq. of an individual mutation at a given vertical position can be measured as the number of reads containing the mutation vs. the total number of reads. Co-existing freq. of two mutations can be measured as the number of reads containing both mutations vs. the total number of reads. The true mutations (in red circles) intend to co-exist on the same reads at much higher frequency than that of random noise mutations (in green stars). B. Based on benchmark dataset 1, all possible pairs of mutationmutation were examined. Each dot represents a pair of both mutations regarding its expected co-existing frequency (vertical freq. of individual mutation 1 x vertical freq. of individual mutation 2) vs. actual observed co-existing frequency (# reads containing the both mutations/# total reads). Red color represents both mutations in the pair as prior known mutations. Blue color represents one mutation in the pair as prior known mutations. Green color represents both mutations in the pair as unexpected mutations.

**Figure 3:**
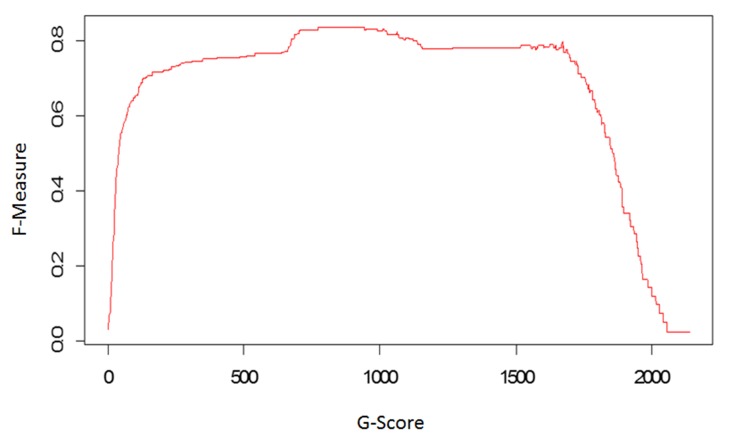
Efficacy of the G test. The sensitivity-PPV curve for the test was examined on the PacBio benchmark data to determine the G statistic that maximizes PPV while achieving perfect sensitivity. The optimal G statistic occurs at a value of 940, corresponding a sensitivity of 1.0 and PPV of 0.695, and an F-measure (harmonic mean of sensitivity and PPV) of 0.82. Plot depicts the F-measure against the full range of G-statistic cutoff values.

A clear phenomenon is that the noises in PacBio reads occur randomly [8]. Thus, for a given pair of two random mutations, the co-occurrence frequency on the same reads is extremely rare (Figure 2). For example, two random variants, with individual noise frequencies respectively of 5% and 2%, are expected to simultaneously occur on the same reads by random chance at 0.1% (5% x 2%). In contrast, the co-occurrence frequencies of mutations are much higher than random chance if the mutations co-exist in biological HIV strain as true mutations. The linkage-frequency strategy developed in this report can easily enlarge discrimination distance between noise and true variant by tens of folds (Figure 2B). The effectiveness of linkage-frequency based noise reduction comes is due to the biological nature of how variants happen, which distinguish between random noise vs. biological true variants during the experimental procedure, rather than simply correcting PacBio noise based on certain pre-defined mathematical distribution model.

The tag-based partition procedure [6] for building final contigs is another important strategy when the reads are noisy. The tag-based partition combined with early linkage-frequency noise reduction allows the analysis to focus on the most likely and important positive spots, therefore, ignoring the remaining unimportant information space during the critical decision-making steps. Our approach largely avoids the distraction of high-level noise on the reads comparing to the typical global alignment and assembly approaches (e.g. overlapping algorithm or de Bruijn Graphic assembly algorithms) [12, 13].

### De novo assembly of HIV quasispecies on the PacBio benchmark datasets

The PacBio HIV benchmark datasets mimic the situation of HIV genetic diversity by admixing two highly close and known HIV strains in different ratios (i.e. 90:10 and 99:1 respectively) [6]. The PacBio benchmark datasets containing ~20,000 CCR reads were applied into our de novo assembly procedure without any other additional information. The de novo assembly procedure produced two final contigs, which are 100% identical to the known HIV strains used for the benchmark experiments (Table 1). In addition, the supporting read counts under each contigs are fairly correlated with the ratios of the benchmark admixture with the detection limit around 0.5-1%. Moreover, in random sampling experiments of simulation (not shown), we found that the minimum number of reads to form a confident contig should be around 50. Thus, in order to achieve the 1% detection limit, we suggest that total number of PacBio reads should exceed 5,000. The results on the well-controlled benchmark datasets suggest that our de novo assembly procedure is able to effectively identify correct contigs for both dominant HIV strain and minor HIV strains up to 0.5-1% detection limit with total number of reads >= 5,000.

### De novo assembly of relapse patient samples leading to early detection of minor drug resistant quansispcies

The real-life HIV patient samples can be more complicated than the benchmark data and therefore more challenging than assembling artificial benchmark data. To further examine the ability of our de novo assembly procedure, two HIV clinical samples from the same patients at two time points (i.e. before and after anti-HIV treatment relapse) had been sequenced using PacBio [6]. As described in previous paper [6], the HIV at time point 1 was well controlled in lower copy number (<3,000 copies/ml) as wild type based on Sanger based sequencing. Conversely, at time point 2 (~ one month later than time 1), the HIV bouncing up (>30,000 copies/ml) indicated the failure of the anti-HIV regiments. In the meantime, an HIV strain containing two strong drug resistant mutations was detected using Sanger sequencing.

Even though Sanger sequencing and Illumina sequencing had been done on the two samples [6], there remained unsolved questions about the situation: 1) Was the HIV drug resistant strain preexistent at time point 1 or exclusively developed between time points 1 and 2? 2) Did the drug resistant mutations occur on the same HIV strains or exclusively on different strains? Answering these questions will help to understand why the HIV drug resistant grew so quickly.

After de novo assembly was applied on the PacBio sequencing of the two samples, the results showed that the patient at time point 1 contained not only dominant WT HIV strains but also minor HIV drug resistant strain at 0.6% level, which later could not be identified by Sanger and Illumina sequencing [6]. Importantly, the minor drug resistant strain in time point 1 is identical to the later dominant drug resistant strain in time point 2. In addition, the two drug resistant mutations are largely on the same HIV strain among the two time points. These PacBio results gave clear answers to previous questions. It may explain why the HIV drug-resistant strain grew so quickly in time point 2, that is, the pre-existing drug resistant strain already existed in time point 1, allowing it to grow quickly by time point 2 under the drug-treatment environment in which WT HIV were largely killed. Moreover, two drug resistant mutations on the same strain can provide much stronger resistance compared to the situation that two drug resistant mutations exist exclusively in different strains. Together, the strong and preexisting HIV resistant strains might be the key reason for anti-HIV treatment relapse. If the PacBio detection information at time point 1 were available in clinic, the early detection of rare but strong HIV drug-resistant strains would be an important indication for clinician to design more appropriate anti-HIV drug cocktails so that the treatment relapse in later time point 2 might be avoided.

## Conclusion

Most of previously developed NGS assembly tools were based on the assumption that the input reads are fairly accurate [12, 13], which is true for the data derived from Sanger or Illumina technologies. When applying these tools on PacBio high-noise reads, their typical global reasoning algorithms (e.g. overlapping assembly and de Bruijn graphic assembly algorithms) are largely driven by noise rather than true signal eventually leading to poor results. In contrast, the proposed de novo assembly procedure is a proactive, positive-focused approach with linkage-frequency noise reduction so that it is more suitable for PacBio high-noise reads. The successful tests on benchmark and real-life patient PacBio datasets suggest that the approach can effectively handle PacBio high-noise reads to accurately assemble dominant and minor HIV quasispecies down to 0.5% without prior knowledge of the sequencing reads. This could be an useful tool to study HIV genetic diversity at explicit quasipecies level, superior to individual mutation level based on Sanger or Illumina technologies currently being used in clinic test.

## Figures and Tables

**Table 1:** *De novo* assembly results on two PacBio HIV benchmark datasets

	Benchmark Admixture		PacBio De Novo Assembly		
Dataset	Strain	Admixture Ratio		# read	Accuracy
Benchmark 90:10	HIV pLN4-3	90%	contig 1	15,020	Exactly matched
	HIV BN10	10%	contig 2	764	Exactly matched
Benchmark 99:1	HIV pLN4-3	99%	contig 1	16,444	Exactly matched
	HIV BN10	1%	contig 2	187	Exactly matched
